# Supplementary material for: Health and economic costs of early and delayed suppression and the unmitigated spread of COVID-19: The case of Australia
Source: PLoS One. 2021 Jun 4;16(6):e0252400. doi: 10.1371/journal.pone.0252400 (PMC8177447; doi:10.1371/journal.pone.0252400)
Supplement: Attachment — Submitted filename: Response to Reviewers.docx [file pone.0252400.s002.docx]

**Responses to Reviewers**

PONE-D-20-18242

Health and Economic Costs of Early, Delayed and No Suppression of COVID-19: The Case of Australia

The authors of this paper sincerely thank the editor and two reviewers for their valuable comments and suggestions. The paper has been greatly improved as a result. Specifically, we have made the following changes in response to the Reviewers’ Comments:

**Reviewer #1**

The authors tackle very relevant and timely issues on the public health and economic burden and benefits of COVID-19 suppression in Australia. The central question is whether the cure (i.e., the lock-down) may be more ‘costly’ in terms of the economy than delayed or no suppression measures. Therefore, the authors constructed a transmission model to fit the observed incidence by estimating epidemiological and suppression parameters. By delaying or relaxing the suppression, the authors calculate the corresponding burden of disease and health cost and make the comparison with the current situation.

I do have some comments on the manuscript and methods.

Comment #1. The transmission model is based on 5 compartments in terms of S-I-Q-R-M. The observed active cases in Australia show a significant drop at the beginning of April and the model fits this behaviour surprisingly well. How did the authors capture this behaviour with a homogeneous mixing model? And even more interesting, how come this drop is not present in their scenario analyses on delayed or no suppression measures?

Response #1: Thank you. We have revised the paper, including the figure label to clarify this point, with the following in mind. The significant drop in active cases at the beginning of April (and another drop in the third week of April) results from a change in the administrative procedure for how a case was classified as ‘recovered’ during March-April 2020. For example, two active cases appear to have recovered (i.e., no longer contagious) on the same day, but were classified as recovered on different days, and some active cases on record appear to have recovered but were not yet classified due to variable administrative procedures early on and/or different testing rates and capacity during a given week – all of which are not predictable outcomes by our epidemiological parameters alone. For this reason, we estimated the number of cases which were classified recovered by combining the epidemiologically estimated total number of cases and the recorded daily recovery rate whenever actual data were available. As there were a spike in the daily recovery classification rates in the beginning and the third week of April 2020, the number of recovery classifications jumped and the number of active cases on record dropped. We have basically included this text verbatim in the revised paper.

Comment #2: The counter-factual scenario “no suppression” assumes that no voluntary behavioural changes occur. As such, “unmitigated spread” would be a better label for this scenario. In addition, the analysis assumes that cost of hospital and ICU admissions can be linearly extrapolated even if the need outreaches the capacity. If hospital beds or ICU’s are not available, other choices will be made which a different impact on the burden of disease and related costs. As such, the statement that the current suppression has saved XXX lives and XXX dollars is controversial. The comparison with a delayed of relaxed suppression seems more informative and reliable.

Response #2: We agree, this is a good suggestion. We have changed ‘no suppression’ to ‘unmitigated spread’ throughout, including in the title of the paper. We also highlight your point that ICU admissions and hospitalisations will reach a limit at some point and thus the comparison with ‘delayed suppression’ may be more informative. The text in section 3.4 reads: “It is important to note that the unmitigated spread scenario assumes no capacity constraints in hospital beds or ICU units, despite the large increase in infections. At some point the capacity of the medical system is breached as indicated in the results below, and a lockdown would likely ensue. In this sense, a comparison of early to delayed suppression is more practical and relevant.”

Comment #3. Not the suppression measures but how the people conceive them and change their social interactions alters the transmission. In addition, the estimated impact in the SIQRM model of gathering bans and business shutdowns is highly correlated and subject of uncertainty. This uncertainty aspect is missing in the numerical calculations.

Response #3: Thank you. We acknowledge both points in our revised paper, at the end of section 3.3 and indicate a related work that considers various social distancing outcomes. The text reads: “Second, note that it is not just the mandated suppression measures that alters transmission, but how the people perceive them and change their social interactions. It is also the case that gathering bans and business shutdowns are highly correlated and subject to uncertainty. We do not account for either in the numerical calculations, given the nature of the compartment model, but we do pick up the point of the impacts of various social distancing outcomes in a related paper (Grafton et al. 2020).”

Comment #4: The impact calculation of patient welfare is not clear to me. They use both the Value of a Statistical Life Year and an age-adjusted Value of Statistical Life. For the latter, they adopt a government estimate of $4.9 million. How is this calculated and which assumptions are made? This is a crucial parameter in a cost-benefit analysis. Please provide more insights on this.

Response #4: (1) Thank you, agreed, this is very important. We use both measures to partly account for age distribution and problems with averaging. VSLY is truncated at an average life expectancy of 82.5 years, which underestimates damages. A-VSL picks up this point and accounts for the fact that most patients who die from COVID in Australia are elderly. (2) We rely on Abelson (2007), the standard reference used by government in Australia to obtain the $4.9 million figure and take greater care to articulate the procedure and assumptions that are adopted here. Please see section 3.4, including the added text: “The estimates in Abelson (2007) are drawn from an extensive meta-analysis of prior VSLY and VSL estimates and includes an overall discussion on the major methods of valuation and empirical results for values of life, health and safety in Australia. It also suggests adjustments for those 70 years and older, although we rely instead on Albertini et al. (2004) as more technically robust. As usual in cost-benefit studies, Abelson (2007) adopts an average WTP value for life, adjusted for age, and thus the VSL is generally held constant regardless of the income of any social group either at any point in time or over time. Most importantly, as conventional, it assumes as a starting point the life of an adult of 40 years of age, likely to live for another 40 years, with again an adjustment for those over 70 years of age.”

Comment #5. Australia has clear guidelines for health technology assessments, which are not fully addressed here (<https://pbac.pbs.gov.au/information/about-the-guidelines.html>) Please make sure all aspects of section 3 “Economic evaluation” are included and reported in the study.

Response #5: Thank you. As you suggested, we have revised our paper to highlight the link you have checked to make certain that we have captured all aspects of section 3 of the guidelines. In particular, as listed in section 3A of the guidelines (Cost-Effectiveness Analysis), our revised paper includes an overview and rationale of the economic evaluation, and a description of the computational method, and population, including model variables and extrapolation. Our paper also includes and discusses health outcomes, health care resource uses and costs, model validation, results for the base-case (our ‘unmitigated spread’ or ‘delayed suppression’ case, depending on context), economic evaluation, as well as uncertainty analysis.

**Reviewer #2**

Comment #6: Abstract:
• Early (actual): not very clear what you mean with actual
• Second sentence is long (3/4 of the abstract) and hard to read; rephrase
• Clarify the methods used

Response #6:

- We have removed the term ‘actual’ from the abstract, it is confusing. Active cases are defined clearly in the paper in any case.
- Thank you, good point. We have rephrased the second sentence, re-worked the abstract and added detail on the methods used.

Comment #7: Introduction
• As of the end of May 2020, the global number of COVID-19 cumulative cases and reported fatalities, respectively, exceed 6 million and 370,000 --> worldwide?
• Update the data -->◊ once accepted use the most recent available data
• (including the President of the United States on 22 March 2020) ◊--> not sure if this adds much. Others including scientists, were also posing this question.
• Several language issues: More costly in terms of the economy ; that we simulate continued until the end of May
• The structure is not logical. Research question comes rather early. Background info is limited. Last paragraph should be moved to method section, results are already given in introduction
• 2. COVID-19 in Australia and policy responses -->◊ include this in introduction section ; this is the background that we need in order to introduce research question
• Information included under heading 2 can also be shortened -->◊ too much detail about progress of pandemic. Main question is, why do we need these analyses?

Response #7:

- Yes, global data available at the time.
- We are going to retain the data relevant to the May 2020 period since it fits the context of the paper and the comparisons we make.
- We have removed the statement from the President, rightly so. Thank you.
- Thank you. We have carefully checked and revised the text.
- Thank you. We have revised the Introduction and, in particular, removed the results from this section.
- It appears to us that Section 2 follows logically from the Introduction, so we have left it as is and we prefer to keep all of the detail (on active case numbers and including the timing of the suppression controls) since it provides the needed context. We have shifted some of the material in the Introduction to this section.

Comment #8: Methods:
• excluded the below-20-year-old population group -->◊ most recent evidence indicated that you should lower the lower bound. Adolescents are also susceptible
• data source not clear
• I do not completely agree with QALY comment, luckily the adjustment for age covers this partially --> discuss this issue in more detail. + effect of age adjustment (scenario analyses of different adjustments)
• Life expectancy for given age should be used
• Why not using the GDP as VSLY?
• You only look at PRO of lock down (effect on COVID deaths) but not at CON (effect of lock down on general population, delay in usual care, increase in more severe diagnosis after lock down, etc…)
• How did you calculate VSLY for recovered patients?
• Why not looking at (modelled) excess mortality compared to previous year?

Response #8:

- We have revised this section to indicate that we have not excluded the under 20-year group in our modelling, while still noting throughout the paper that almost all of the fatalities are over 60 years old.
- We have double-checked to make certain that the references are clear. Data on all compartment categories is official government data, as indicated, mostly drawn from Covid-19-Data (2020).
- Thank you, we have added further discussion of ‘age adjustments’ to the welfare measures throughout but left the calculations as a .70 reduction for those over 70 years unchanged for the adjusted value of a statistical life, following Abelson (2007). Please also see section 3.4, including the added text: “The estimates in Abelson (2007) are drawn from an extensive meta-analysis of prior VSLY and VSL estimates and includes an overall discussion on the major methods of valuation and empirical results for values of life, health and safety in Australia. It also suggests adjustments for those 70 years and older, although we rely instead on Albertini et al. (2004) as more technically robust. As usual in cost-benefit studies, Abelson (2007) adopts an average WTP value for life, adjusted for age, and thus the VSL is generally held constant regardless of the income of any social group either at any point in time or over time. Most importantly, as conventional, it assumes as a starting point the life of an adult of 40 years of age, likely to live for another 40 years, with again an adjustment for those over 70 years of age.”
- We only have life expectancy for a given age as it relates to average life expectancy. We have clarified this point.
- We have measures for the fall in GDP but also included VSLY and A-VSL because these are standard measures.
- We have briefly highlighted the negative impacts of ‘lockdown’, while still noting that many of these impacts were mitigated through government assistance programs in the Discussion section. We have also re-emphasized that morbidity effects from COVID are not included. Unfortunately, there is no available quantitative measures to be able to include in our modelling.
- Thank you. We measure VSLY only for mortalities and account for hospitalisation and other costs for those patients who die and ultimately recover separately. We have clarified this point. We do account for the average number of sick days of patients who do recover, specified at 18.5 days on average.
- Thank you. We have provided additional references to acknowledge this point, just before section 4.1.

Comment #9: Results
• It would be very informative to see the results of individual different measures included in lock down--> closure of schools, shut down of non-essential business, social distancing.

Response #9: Agreed, unfortunately there is insufficient data for us to include in our modelling.

Comment #10: Discussion
• The discussion lacks comparison with other studies. It is merely a summary of own findings.
• Problem with +82 year olds could be solve by using life expectancy at given age
• What was the influence of time? We see that the number of deaths is now decreasing, even though less measures are in place compared to lock down? How should we look at upcoming months? Go hard, go early? Of a more adapted scenario?

Response #10:

- Thank you. To the best of our knowledge there is no published material or pre-print that combines an epidemiological model with the impacts on the economy from various mandated suppression measures (including the case of unmitigated spread) for Australia – save for a non-technical discussion in Group of Eight Australia (2020) and also Grafton et al. (2020) which are cited in the revised manuscript.
- We only have access to average life expectancy but note your suggestion/qualification in the revised paper.
- Thank you. We contend (based on our modelling results) that a ‘go hard, go early’ is the preferred public policy choice.
