# Supplementary material for: Health and economic costs of early and delayed suppression and the unmitigated spread of COVID-19: The case of Australia
Source: PLoS One. 2021 Jun 4;16(6):e0252400. doi: 10.1371/journal.pone.0252400 (PMC8177447; doi:10.1371/journal.pone.0252400)
Supplement: Attachment — Submitted filename: Response to Reviewers.docx [file pone.0252400.s003.docx]

**Response to Reviewer(s)**

Thanks again to Reviewer #1 for providing very helpful comments. We greatly appreciate the time she/he has spent with our paper! Our responses are below.

*Reviewer #1: The authors improved their manuscript and resolved many issues from both reviewers. Thank you for your gratitude and by taking our feedback seriously. The current message is more nuanced and thus informative, in my opinion. I still have a few comments.*

1. *The authors replied on my question (#1) why the reported cases dropped at the beginning of April. The added text (line 175-180) is rather long and difficult to read. However, changes in reporting strategy and other administrative issues have taken place worldwide and interfere with modelling, though my question was, “how did your SIQRM model capture this sudden drop”? What kind of temporal parameter(s) did you include to capture this observed behavior? How did you inform these parameters? And what consequences does this additional temporal “model intervention” has for making predictions?*

**Response**: Thank you for this point. We have clarified the text to read: ‘There are two additional points to make. First, note that the significant drop in active cases at the beginning of April (and another drop in the third week of April) results from a spike in the number of cases which were classified as ‘recovered’-- not all of which are predictable by epidemiological parameters alone because how a case was classified as recovered could depend on administrative and reporting procedures. For this reason, during the period where actual data was used to estimate the epidemiological model (i.e., March 1^st^ to April 20^th^, 2020), we combine the fitted total number of cases (using epidemiological parameters only) and the daily recovery classification rate calculated from the actual data to estimate the number of cases which were reported as recovered. To test the prediction capacity or the accuracy of the epidemiological model, we used a period where the data was ‘new’ to the model (i.e., April 21^st^ – May 11^th^, 2020), and in this testing situation, the number of ‘recovered’ cases was predicted by combining the epidemiologically predicted total number of cases with the average number of sick days of patients who recover, i.e., approximately 18.5 days (Covid19-Data, 2020).’

2. *The counterfactual scenario on “no suppression” is well explained in the revised manuscript, though the additional note that “a lockdown would likely ensure” seems to undermine the scenario. This is stated at line 206 and 440. In my comment #2, I referred to “a total breakdown of the medical system will be even more deadly than the estimates, since there are not enough e.g. ICU’s to save severe cases and all other care is not possible. The costs are uncertain, since you cannot pay for ICU’s or ventilators that are not there. Many patients will not be admitted to ICU, though you include unlimited ICU costs”. The bottom line of this scenario, is that there is no lockdown.*

**Response**: Understood, thank you. Our only point is that if the capacity of the medial system is exceeded the government position would indeed be to enforce a severe lockdown. Our counterfactual ignores this, of course. We have adjusted the text (near line 206) to clarify the point.

3. *On line 211, the authors explicitly challenge the usefulness of QALYs for cost-effectiveness comparisons among alternative public health responses. Unless they have a vast amount of literature to backup this claim, I would just mention that the analysis is based on life-years-lost and not on QALYs, in line with the Guidelines. On line 244, the authors report their scaling of the VSL of 4.9million dollar by 0.7 for people over 70 years of age. This is not clear to me. So, 70-year olds represents only/still 30% of their monetary value to society? Please explain this more for the reader.*

**Response***:* Thank you. We have removed this part of the discussion on QALYs.

**Response**: We have kept the text as is, although certainly acknowledge that the value of a statistical life can be calculated in different ways and, depending on the method used, may vary with a person's age. If based on future earnings, then persons who are young adults may have a higher relative VSL to an older demographic. If based on ability and willingness to pay (WTP) measures, middle-aged or older persons may have a higher relative VSL to a younger demographic. We followed the findings of Alberini et al. (2004) who found weak support that WTP declines with age, and only for older respondents (aged 70 and above) in their surveys of two populations (one in Canada and one in the US). They found that their Canadian survey respondents over the age of 70 were willing to pay about one-third less than their younger counterparts to reduce their risk of dying by 5 in 1000 over the next 10 years. Thus, we adopted the heuristic to reduce VSL of persons over 70 years of age by 0.30 noting that a similar heuristic (0.75 X VSL for persons 65 years and older) has previously been employed by Health Canada.

4. *The impact of mandated suppression on tourism seems not completely fair. It is sure that the measures in place prevented people from coming to Australia, and this has an economic cost. However, this pandemic also affects outgoing passenger flows in other countries. Even without lockdown, there will be economic loses for tourism. Hence, accounting the full economic loss for tourism to the lockdown requires some remarks.*

**Response**: We agree. The decline in tourism to Australia is primarily a result of the supervised 14-days quarantine on all arrivals, the cost of which must be paid by those arriving in Australia. This is approximately $3,500 per person.  Supervised quarantine requires a large number of quarantine workers and suitable rooms to isolate travellers. Resourcing constraints, particularly a sufficient number of beds in appropriate hotels, has also meant the that the number of people permitted to come to Australia is about 20,000 per month. (see [https://www.abs.gov.au/statistics/industry/tourism-and-transport/overseas-arrivals-and-departures-australia/latest-release](https://protect-au.mimecast.com/s/_dqDCYWLOxhLORoY2F0_tgm?domain=abs.gov.au)) while pre-COVID in 2019 arrivals were, on average, 700,000 per month. Further, these constraints on arrivals continue despite the fact that the national lockdown ended in May 2020.  In sum, attributing the full loss in tourism to the lockdown, as we have done, overestimates the lockdown cost.  Thus, in the revised version we state: 'In summary, we attribute all the costs of reduction in international tourism to the lockdown. This overstates the costs of lockdown because a decline in international tourism would have occurred even in the absence of stringent social distancing. This is because of Australia's international border controls limit the number of arrivals into Australia and requires supervised quarantine of 14 days. These international border controls have been continuously maintained even when national lockdown ended in May 2020.' (line 376)

5. Line 421 states that “delays in suppression provide no economic gain, but increased fatalities and …). This gives the impression that the economic cost of the suppression does not depend on the timing. Only the fatalities are impacted by the timing of the suppression measures. Would it be possible to elaborate more on this?

**Response**: Thanks for this. We have added your qualification.

6. The following statement in the abstract and partly repeated in the discussion (line 449) is not clear to me: "We also find that using an equivalent VSLY welfare loss from fatalities to estimated GDP losses, drawn from survey data and our own estimates of the impact of suppression measures on the economy, means that for early suppression not to be the preferred strategy requires that Australians prefer more than 12,500 - 30,000 deaths, depending on the fatality rate, to the economy costs of early mandated suppression.” This reads like Australians have to choose between early suppression or 12500+ deaths. This seems not the take-home message from this manuscript. What about the nuance between delayed suppression? In addition, this assumes a fixed willingness-to-pay which is rather a complex concept and a subject for discussion.

**Response**: Yes, agreed, good point. The use of ‘Australians prefer’ is clearly bad form. We’ve reworded the material (in the abstract and conclusion) to pick this up and the point on unmitigated spread, indicating just model results. The abstract reads ‘ … from survey data and our own estimates of the impact of suppression measures on the economy, means that for early suppression not to be the preferred strategy requires that Australia would have to incur more than 12,500 - 30,000 deaths, depending on the fatality rate with unmitigated spread, to the economy costs of early mandated suppression. We also find that early rather than delayed mandated suppression imposes much lower economy and health costs and conclude that in high-income countries, like Australia, a ‘go early, go hard’ strategy to suppress COVID-19 results in the lowest estimated public health and economy costs.’

We have left aside your point of assuming a fixed willingness to pay as indeed too complex in this version of the paper – something for further research.

7. *Minor comments
- “Active cases” is rather uncommon. The number of active cases over time can be indicated by the prevalence.*

**Response**: We have kept it as is, as standard practice in Australia at the time. Active cases are the numbers of individuals identified and in quarantine.

*- Line 60, how did the authors calculated the daily growth rate?*

**Response**: We have clarified this in the text to read: ‘As of 1^st^ June 2020, Australian mandated suppression measures drastically reduced community transmission of the virus and the daily growth rate (the daily increase in the total number of cases over the total number of cases, on a three-day average) declined from around 25%, with 268 new recorded cases on 22^nd^ March, to a daily growth rate of 0.26% and 11 new recorded cases on 30^th^ May 2020 (Covid19-Data, 2020).’

- *The manuscript contains ‘Error! Reference source not found”*

**Response**: Repaired. It came through this way in the PDF but is fine in the Word document. In any case, we have re-entered the source.

- *Line 325: what do the authors mean with “Some 84 percent of businesses…” ?*

**Response**: It is 84%. We have removed the ‘some’.

*- Line 344 misses the Figure number*

**Response**: Repaired, thanks.

*- Line 432, what do the authors mean with “the spread of COVID-19” ?*

**Response***:* Thanks, we have removed this sentence. It wasn’t needed.

*- Line 471, please rephrase “public health costs and economy costs”.*

**Response**: Repaired, thanks.

*- In the equations, the letter “T” is re-used in different equations. This is confusing.*

**Response**: Thank you for this point. We have revised the paper to clarify that $T$ (without the superscript) is the total number of observed cases, and the transpose matrix operator, which was also denoted as $T,$ is now denoted as $Tr$.

- *Line 105, R0 is defined as the basic reproduction number and not the “initial” reproduction number.*

**Response**: We have revised this sentence as suggested, thank you.

- *The manuscript does not include any comment or remark that this model is an abstraction of the incubation, symptomatic, pre-symptomatic and asymptomatic infection stages. The model does not include any time lag between infection and infectiousness, which has impact on the timing of the predicted epidemic. The model structure is clearly stated and fits the purpose, though a remark on this issue would put the analyses in perspective.*

**Response**: Thank you. We have included a revised version of your qualifier in the technical section (near line 101), which reads as ‘It is important to note that the model, although fit for purpose, is an abstraction of the relevant infection stages and does not include any explicit time lag between infection and infectiousness, which has impact on the practical timing of the predicted epidemic in Australia.’
